# Healthcare providers' intention to leave their jobs during COVID‐19 pandemic: A cross‐sectional study

**DOI:** 10.1002/hsr2.859

**Published:** 2022-10-04

**Authors:** Mohammad M. Alnaeem, Ayman M. Hamdan‐Mansour, Abdulqadir J. Nashwan, Alaa Abuatallah, Mahmoud Al‐Hussami

**Affiliations:** ^1^ School of Nursing Al‐Zaytoonah University of Jordan Amman Jordan; ^2^ School of Nursing The University of Jordan Amman Jordan; ^3^ Nursing for Education & Practice Development Hamad Medical Corporation Doha Qatar

**Keywords:** COVID‐19 pandemic, healthcare providers, intention to leave job

## Abstract

**Background and Aims:**

During the coronavirus pandemic (COVID‐19), healthcare providers confronted risks of disease transmission to themselves and their family members, resulting in physical and psychological burdens. This might affect their decisions to leave their jobs temporarily or permanently, fearing infection and protecting their families. This study examined the factors related to the intention to leave a job among healthcare providers during the COVID‐19 pandemic in Jordan.

**Methods:**

A cross‐sectional correlational design was used to collect data using a convenience sample of 557 healthcare providers working in different sectors across Jordan. Data were collected using a self‐administered questionnaire about the intention to leave jobs during the pandemic.

**Results:**

The sample included 368 females (63.8%) and 209 males (36.6%) participants. The mean age of participants was 30.8 years (SD = 6.65). Differences found in intention to leave job during COVID‐19 in relation to age (*t* = 2.60, *p* < 0.05), gender (*X* ^2^ = 4.25, *p* < 0.001), and marital status (*X* ^2^ = 18.2, *p* < 0.001). Participants with a high risk of exposure to COVID‐19 and who experienced higher workloads had higher scores of intention to leave their job during COVID‐19, while being married had lower scores.

**Conclusions:**

Policy‐makers need to pay attention to young and single healthcare providers during the COVID‐19 pandemic to prevent them leave their job. Crucial guidelines for managing workload during the COVID‐19 pandemic are needed. Policy‐makers during pandemics have to protect healthcare providers who feel they are at high risk of infection.

## 1 BACKGROUND

The coronavirus disease (COVID‐19) pandemic became a universal health issue that alarmed the healthcare systems worldwide.^1^, ^2^, ^3^ Along with a rapid and unexpected increase in the incidence of COVID‐19, countries were forced to develop and adopt massive public health restrictions to ensure protection and minimize the impact of the COVID‐19 pandemic. It had negative impacts on several domains, including but not limited to educational, financial, and entrepreneurship globally.^4^, ^5^, ^6^ Healthcare providers (HCPs), whom are called to be at the frontline of COVID‐19, are the most affected and are vulnerable to various forms of consequences of COVID‐19. Healthcare systems, therefore, were required to balance maintaining a safe and healthy work environment for HCPs while providing a high quality of healthcare to infected people. Nevertheless, although HCPs have used strictly personal protective equipment, they were concerned about being infected and being sources of disease transmission to their beloved ones.^7^ This has provoked the attention of healthcare systems to psychosocial health needs and concerns of HCPs caring for individuals with COVID‐19. The COVID‐19 pandemic has affected the daily life activities and biopsychosocial health status of HCPs.^8^, ^9^, ^10^, ^11^

The literature adequately addressed the psychological and mental health consequences of caring for individuals with COVID‐19. Generally, studies showed that HCPs are 10‐folds at risk of COVID‐19 compared to the general population^12^ and suffer various forms of psychological disturbances, including depression, anxiety, psychological distress, and sleep disturbances.^13^ HCPs were also suffering from burden and workload, causing them further psychical fatigue.^7^, ^14^, ^15^, ^16^ Such a situation, although it seems critical to HCPs, fearing infection, being sources of infection, and suffering social discrimination and stigma due to caring of individuals with COVID‐19, it forced HCPs to socially isolate themselves caring further psychosocial disturbances.^13^, ^17^ Besides the extension of the world emergency to combat COVID‐19, the short and long‐term consequences have contributed to job dissatisfaction, burnout, and intention to leave the job.^18^ One factor found to contribute to employee decision to leave their jobs in healthcare settings is their perception of an unsafe working environment.^19^ Such feeling is well enhanced with the chaos that has been witnessed during the few months of the outbreak of COVID‐19.

The panic public response and the lack of resources and workforce have a significant role in increasing fear and worries among HCPs that may force many of them to leave the job, fearing infection and to look after their families. Before COVID‐19, stressful working conditions, work overload, and burden were factors associated with the intention to leave their job in spite of their feeling of although a safe working environment and sufficient resources.^20^, ^21^ This would speculate that with the outbreak of COVID‐19 pandemic that associated with shortage of workforce and resources, HCPs would think more seriously to leave their jobs. As aforementioned, HCPs were suffering stress, anxiety, and depression similar to general population and further were at higher risk to get infected with COVID‐19 due to nature of their work. Factors such as commitment and work conscience, fear of family infection, fear of shortage of protective equipment, and organizational factors were affecting nurses' intentions to leave or stay in their profession during the COVID‐19 pandemic. A recent study revealed that clinical nurses and nurse leaders who reported higher levels of contact with and management of individuals with COVID‐19 had higher intent to leave their jobs.^22^ While such topic was important and significant issue for discussion, few studies attempted to address such topic in low‐income countries such as Jordan. Jordan has limited resources and the healthcare systems and infrastructure is still developing which may compromise the quality of care and the HCPs willing to stay in their jobs. This is one study that attempt to understand the impact of COVID‐19 on HCPs in terms of intention to leave their jobs due to the fact that burnout and intention to leave job would affect directly quality of care provided and considered a threat to stability of healthcare systems. Therefore, the purpose of this study is to examine the intention to leave the job during the COVID‐19 pandemic among HCPs in Jordan. The research questions are:

1. What are the factors associated with HCPs' intention to leave their job seriously?
2. What are the factors associated with HCPs' perception of intent to leave their job seriously?

## 2 METHODS

### 2.1 Design

A cross‐sectional correlational study was conducted during the COVID‐19 pandemic using an online survey between October 5 and November 15, 2020.

### 2.2 Sample

A convenience sampling technique was used to select HCPs caring for patients diagnosed with COVID‐19. The HCPs who provided care directly to patients with COVID‐19 and had internet access and skills to fill out the survey online were included in the study. No exclusion criteria were used to maximize participation.

### 2.3 Settings

As the nature of the online survey, this study was conducted via a structured questionnaire sent online through a social network and email to accessible HCPs from the Jordanian healthcare sector, including various governmental and teaching hospitals. These hospitals extended over all regions (north, middle, east) of Jordan and provided care for the majority of patients in Jordan (67%).

### 2.4 Measurements

Data was collected using an online platform of an Arabic self‐administered survey. WHO guidelines for translation and tool adaptation were used to translate the instruments that are unavailable in Arabic. The instruments have been evaluated for validity using the face and content validity methods through a panel of experts. The WHO guidelines were used and followed to reach the final draft of the translated versions of the survey. The survey in the current study has three parts. The first part is related to socio‐demographic data (six items), including age, gender, marital status, job description, length of experience, and previous experience with patients with serious diseases. The second part consists of two items to assess the serious of HCPs' intention to leave the job. Item 1 was “Looking for another job or considering resigning because of the risk of COVID‐19,” and item 2 was “Considered that should not care for patients with COVID‐19.” Each item has two scores; score “1” if the answer is “yes” and score “0” if the answer is “No.” If participants answered both questions with “Yes,” they would consider leaving the job seriously.

The third part is about perceived leaving the job during the COVID‐19 pandemic, which included 32 items. The 32 items were grouped into seven subscales: organizational support (six items), perceived risk of contracting COVID‐19 (seven items), workload and stress (five items), social relationships (four items), emotional support (five items), the perceived fatality of COVID‐19 (three items), and personal protective equipment (two items).^23^ Each item scored on a 5‐point Likert scale where the score of “1” reflected strongly disagree, and “5” strongly agree. A high score means a high level of perception to leave HCPs the job. The Cronbach alphas for each subscale varied from 0.73 to 0.86, revealing accepted internal consistency.

### 2.5 Ethical Issues/Statement

The Institutional Review Board at Prince Hamza Hospital has approved this study. The current study followed the Declaration of Helsinki provisions, and all participants provided informed consent. The permission to use and translate copyrighted instruments has been received from the original author. No approval from hospitals was required according to the nature of the online study. The study was conducted following the EQUATOR research reporting checklist; (STROBE checklist) for cross‐sectional research. It was uploaded to the online system.

### 2.6 Data collection

After checking the instruments for reliability and validity, minor changes were performed based on expert panel recommendations. The online pilot survey was then carried out on 25 randomly selected HCPs to assess the survey's clarity, relevance, acceptability, feasibility, and time needed. Improvements were made to facilitate better comprehension and organize the survey items before the last draft. The time needs to complete the survey is less than 15 min. These participants were excluded from the study.

The target population was accessed through a URL link posted in a Facebook group established in October 2018, publishing the latest news and regulations about healthcare services and illness statistics updates. The Facebook group includes more than 400 thousand active members of HCPs all over the healthcare institutions in Jordan. In addition, the questionnaire was sent via email. After entering all items into the software, the URL link was sent and opened on October 5 for 15 days. The software password is kept only with the primary author to anonymize participants' confidentiality and information. The participants voluntarily enrolled in this study without offering any monetary incentive.

The online informed consent was appended to the survey on the front page. Once the participant receives and clicks the link, it is automatically directed toward the front page. The front page included information to assure the participants of their right to voluntary participation and their right to withdraw at any time. Interested ones were asked to sign the consent letter and then move them directly to the survey included in the first page before the socio‐demographic information. The website views the remaining questions sequentially on an average of 9–10 items per page. Also, to ensure the quality of collected data, a unique number was assigned for each participant to avoid multiple responses from the same person. Furthermore, the identifiable information provided in the socio‐demographic section was separated in analysis from the other variables of the study. Finally, on the platform page was a list including participants' codes, address, time of completion survey, and the device used to answer the items (e.g., iPhone, laptop, Samsung. etc.).

### 2.7 Data analysis

The IBM‐SPSS (V. 25.0) software was used to analyze the data. Descriptive statistics using the central tendency and dispersion measures were used to describe the study's variables. Frequencies and percentages were used to describe the categorical variables. The chi‐square test and independent *t*‐test were utilized to assess the difference between respondent characteristics and serious consideration of leaving the job. Multivariate logistic regression was conducted to determine the predictive factors associated with HCPs considering leaving the job. The relationship between the perception of HCPs to leave the job and participants' characteristics was assessed using analysis of variance (ANOVA) or *t*‐test for independent categorical variables and Pearson correlation coefficient for the continuous variables.

## 3 RESULTS

### 3.1 Socio‐demographic data

Of 798 HCPs interested in participating, 703 (88.1%) completed the survey. About 126 surveys were excluded because they were incomplete (79 surveys had at least one part of the survey completely unfilled and 47 surveys had incomplete answers for half of the items in two parts or more). The remaining 577 surveys represented the final sample and were included in the analysis. Of 577 HCPs, 368 were females (63.8%), and 209 were males (36.6%). The mean age of participants was 30.8 years (SD = 6.65). More than half of the participants were married (*n* = 326, 56.5%). Most of the participants (*n* = 502, 87%) were nurses who were directly involved in providing direct care for patients with COVID‐19. Despite 30.5% of participants having less than 5 years of experience, 64.1% have previously cared for patients with infectious diseases (Table 1).

**Table 1:** Demographic and work characteristics of the participants

Characteristic	No.	%
Age		
(mean ± SD)	30.8 (6.65)	
Min–Max	21–53	
Gender		
Male	209	36.3
Female	368	63.8
Marital status		
Single	251	43.5
Married	326	56.5
Job description		
Nurses	502	87.0
Physicians	34	5.9
Others	41	7.1
Years of experience in current job		
<5 years	176	30.5
5–10 years	194	33.6
>10 years	207	35.9
Ever cared for patients with serious infectious disease		
Yes	370	64.1
No	207	35.9

#### 3.1.1 HCPs' serious consideration of leaving the job

The item analysis showed that 26.2% of the respondents agree with the statement “I feel that I should not be looking after patients with COVID‐19,” and 36.7% agree with the statement “I am looking for another job or considering resigning because of the risk,” see Table 2. Also, those who agreed with both statements accounted for 61 participants and considered seriously leaving the job (10.6%). In addition, the chi‐square results revealed that gender was significantly related to intent to avoid caring for patients with COVID‐19 (*χ* ^2^ = 2.94, *p* < 0.05) and seriously considering leaving the job (*χ* ^2^ = 4.25, *p* < 0.05). Similarly, marital status was significantly associated with intent to avoid caring for patients with COVID‐19 (*χ* ^2^ = 16.4, *p* < 0.001) and seriously considering leaving the job (*χ* ^2^ = 18.2, *p* < 0.001). Moreover, participants' age was related to looking for another job and seriously considering leaving the job (*t* = 3.7, *p* < 0.001). In contrast, the length of experience was related to looking for another job (*χ* ^2^ = 24.2, *p* < 0.001).

**Table 2:** Differences between healthcare providers' characteristics and serious consideration of leaving their job (*N* = 577)

Consideration of leaving their job	Looking for another job or considering resigning because of the risk of COVID‐19 (*n* = 151)	Considered that should not care for patients with COVID‐19 (*n* = 212)	Seriously considering leaving the job (*n* = 61)
Age	3.70** ^,^ a	0.04^a^	2.60* ^,^ a
Gender	0.25^b^	2.94* ^,^ b	4.25* ^,^ b
Marital status	2.13^b^	16.4** ^,^ b	18.2** ^,^ b
Job description	5.34	7.13^b^	3.66^b^
Length of experience	24.2** ^,^ b	0.06^b^	4.40^b^
Previous experience	0.14^b^	5.46* ^,^ b	1.35^b^

*Note*: *p < 0.05, **p < 0.01.

^a^ *t*‐test.

^b^ Chi‐square test.

#### 3.1.2 Factors affecting HCP's consideration of leaving the job

Factors contributing to leaving the job were assessed using multivariate logistic regression (Table 3). The logistic regression model was statistically significant, *χ* ^2^ (15) = 148.74, *p* < 0.001. Married HCPs have a significantly lower consideration for leaving the job seriously than single HCPs (*B* = 1.21, odd ratio = 0.29, *p* < 0.01). About the perception of HCPs of leaving the job, those who feel that the job puts them at great risk of COVID‐19 (*B* = 1.57, odd ratio = 0.488) and experience a high workload (*B* = 3.28, odd ratio = 26.6) were significantly associated with seriously considering leaving the job (*p* < 0.01). Moreover, HCPs who reported less seriously considering leaving the job perceived that people recently avoided them because of the job. Besides, less appreciation from their organization (*B* = 0.73, odd ratio =  5.17) and believing that they could die from COVID‐19 more than from cancer (*B* = 2.28, odd ratio = 42.9) were significant predictors for more considering leaving the job.

**Table 3:** Predictive factors for healthcare providers' consideration of leaving their job

Characteristic	B coefficient	SE	Odds ratio	95% CI (lower‐upper)	*p*‐Value
Age	−0.057	0.039	0.94	0.88−1.02	0.143
Gender (Male)	−0.117	0.346	0.89	0.45−1.75	0.735
Marital status (Single)	−1.21	0.425	0.29	0.129−0.686	0.004**
Job description					
Physicians	−0.840	0.799	0.43	0.09−2.07	0.293
Nurses	−0.249	.522	0.78	0.28‐15.5	0.47
Cared for patients with serious infections	0.235	0.319	1.27	0.68−2.36	0.461
Availability of clear policies and protocols	−0.310	0.341	0.73	0.38−1.43	0.364
Great risk of exposure to COVID‐19	1.57	0.488	4.78	1.84−12.5	0.001**
Workload	3.28	0.539	26.6	9.23−76.4	0.0001**
People avoid me because of my job	−3.15	0.553	32.5	0.014−0.126	0.0001**
Appreciated from organization	−0.73	0.319	5.17	0.259−0.905	0.023*
Dying from COVID‐19 is higher than cancer	−2.28	0.394	42.9	0.035−0.164	0.0001**
Availability of protective measures	− 0.207	0.321	0.415	0.433−1.53	0.519

*Note*: *R* ^2^ = 0.397. *p < 0.05, **p < 0.01.

#### 3.1.3 Perception of HCPs leaving the job

The relationship between the perception of HCPs of leaving the job and socio‐demographic variables is described in Table 4. Younger (*r* = −0.21, *p* < 0.001), and male participants of HCPs (*t* = −4.45, *p* < 0.001) feel at higher risk of being infected with COVID‐19. Also, male HCPs (*t* = −6.56, *p* < 0.001) and those with long experience (*F* = 3.17, *p* < 0.05) experienced more workload and stress during the COVID‐19 pandemic. Older HCPs (*r* = 0.09, *p* < 0.05) with long experience in the job (*F* = 3.09, *p* < 0.05) and previous experience caring for patients during a pandemic (*t* = −4.14, *p* < 0.001) noted more social relationships during the COVID‐19 pandemic. Also, younger (*r* = −0.11, *p* < 0.001) and married HCPs and those with long professional experience (*F* = 15.5, *p* < 0.001, *F* = 7.96, *p* < 0.001; respectively) stated more emotional support than others. Nevertheless, the younger, married HCPs and those without previous experience in caring for patients with infectious diseases reported the possibility of fatality from COVID‐19 more than other illnesses. All socio‐demographic variables were significantly associated with the perception of HCPs about the adequacy of personal protective equipment in their departments.

**Table 4:** Relationship between the perception of healthcare providers about leaving their job and socio‐demographic variables

Variables	Organizational support	Perceived risk of contracting COVID‐19	Workload and stress	Social relationships	Emotional support	Perceived fatality of COVID‐19	Personal protective equipment
Age	0.04	−0.21** ^,^ a	−0.04	0.09* ^,^ a	−0.11** ^,^ a	−0.18** ^,^ a	−0.19** ^,^ a
Gender	0.41	−4.45** ^,^ b	−6.56** ^,^ b	−1.80	0.48	−5.65** ^,^ b	−4.82** ^,^ b
Marital status	22.2** ^,^ c	0.64	1.87	2.61	15.5** ^,^ c	3.92* ^,^ c	37.1** ^,^ c
Job description	0.69	1.32	0.42	1.07	0.8	2.26	3.05* ^,^ c
Length of experience	0.61	1.78	3.17* ^,^ c	3.09* ^,^ c	7.96** ^,^ c	0.78	19.4** ^,^ c
Previous experience	−1.36	−0.69	−1.42	−4.14** ^,^ b	1.09	−2.25* ^,^ b	−2.26* ^,^ b

*Note*: *p < 0.05, **p < 0.01.

^a^ Pearson correlation.

^b^ *t*‐test.

^c^ Analysis of variance.

## 4 DISCUSSION

The pandemic of COVID‐19 has affected the mental and psychosocial well‐being of individuals and HCPs, as well; work overload, distress related to COVID‐19, and fear of infection and being a source of infection were the most reported factors that influenced HCPs.^13^, ^17^, ^24^ Such working conditions are assumed to influence the HCPs' decision to leave the job seriously. While HCPs have the right to leave the unsafe workplace based on their beliefs if the job is safe or not,^19^ their decision might not simply be possible and easy process due to ethical and legislative issues that might interfere with their independent willing to make the decision. This study, in general, found that a significant proportion of the HCPs were seriously thinking of leaving their jobs. This could be related to the fact that the confabulated information disseminated by media and the social discrimination, besides the extensive workload might have contributed to such a decision.^17^, ^24^ Although the figures seem to be not great in terms of magnitude (10%); however, and due to the shortage of HCPs and the emergency situation globally, the figure was alarming and threatening directly the quality and ability of healthcare systems to carry out their responsibilities towards caring of individuals with COVID‐19.

Nevertheless, the finding in this study was supporting previous reports that almost equal percentages of nurses had an intention to leave their jobs during the pandemic of COVID‐19.^22^ We also found that HCPs with fewer years of experience or those without past experience caring for individuals with infectious diseases were more likely to consider leaving the job during COVID‐19. Such findings sustain previously reported findings and positively connect years of experience to intention to stay in the job.^25^ In other words, HCPs with fewer years of experience might be unable to adapt to the intense job requirement and feel incompetent due to a lack of experience in managing such cases and intense death scenarios. However, those with long years of experience are willing to use their past experiences and willing to adapt more successfully to the up‐to‐date requirement and needs and respond effectively to the emergent healthcare need of their patients. In particular, it is assumed that novel HCPs are more apt to respond negatively to the panic response of media, stigma, and negative public response to COVID‐19 than those with long years of experience.

During the COVID‐19 pandemic, other factors are associated with leaving the HCPs their jobs, such as the availability of personal protective equipment. Notably, the low availability of protective equipment probably increased the risk of leaving the HCPs their jobs.^26^ However, the results indicated that personal protective gear's effectiveness was not perceived as a significant factor for the intention to leave the job; this could be related to the availability of clear protocols and resources efficiently at work. The highest preparedness to deal with the COVID‐19 pandemic was associated with sufficient protocol availability to approach patients and hospital personal protective measures.^27^

COVID‐19 can easily spread to individuals close to HCPs as patients, families, and friends.^28^ Therefore, HCPs faced inescapable tension and fear, especially about the probability of transmitting COVID‐19 to their families. A recent qualitative study conducted to explore the intentions of nurses to leave or stay in their profession after the COVID‐19 pandemic revealed that many nurses explain the fear of infecting their families and lack of protective equipment at the hospital as the main sources of their fears and worries.^29^ Studies have also suggested managing the workforce‐related stressors and building a healthy work environment to decrease or ease the HCPs' burnout. A recent study found that nurses who directly interact with COVID‐19 patients were more worried about being infected and have higher job stress and burnout levels than nurses who are not.^30^ Similar findings were also found among HCPs in China and United States.^31^, ^32^, ^33^ The current findings indicated that HCPs who intended to stay at the job were more than those who had a perception to leave the job seriously. This could be related to several factors, including organizational commitment and a sense of self‐achievement.^34^, ^35^ Moreover, the younger and single HCPs more intent to leave the job seriously during the COVID‐19 pandemic than others. Similar findings of recent studies supported these findings.^36^, ^37^ Also, this study confirms that male HCPs intent to leave their profession than females; these findings are congruent with a previous conclusion.^38^ On the other hand, some studies found no relationship between the intent HCPs to leave the job and their socio‐demographic variables.^39^, ^40^

## 5 IMPLICATIONS OF THE STUDY

The current study may shed light on some clinical practice and leadership implications, such as presenting clear policies that aim to protect HCPs during such pandemics and offering support from leaders. These points could increase their self‐confidence, improve their morale, motivate them, and decrease their intention to leave the job. Offering personal protective measures in healthcare settings will give the HCPs a sense of safety and maximize teamwork that minimizes stress. Promoting psychosocial support for HCPs from their organizations and the public will enhance their satisfaction and minimize their fear, resulting in low intention to leave the job.

Considering that participants were enrolled from various public hospitals in the region, this could reflect the strength of the study and thus could support the generalizability of the findings to Jordan. Similarly, the study considered a clustering effect because the data were collected from different regions. However, this study had some limitations related to the use of an online cross‐sectional survey that might affect the attrition rate of the participants. In this regard, many surveys were excluded due to missing data that could introduce potential bias and affect the study's findings. In addition, missing some confounding factors such as the specialty of the physicians, professional level, and daily working hours could all affect the participants' intention to leave job.

## 6 CONCLUSIONS

This study highlighted that HCPs with a high risk of exposure to COVID‐19 and who experienced more workload were seriously considering leaving their jobs. At the same time, married participants have lower consideration for leaving their jobs. Policy‐makers need to pay attention to young and single HCPs during the COVID‐19 pandemic to prevent them from leaving their job. Clear guidelines for managing workload during the COVID‐19 pandemic are needed. Furthermore, as the global risk of COVID‐19 infection remains high and HCPs in several countries could be confronted with similar challenges, all possible psychosocial and work‐related factors should be urgently identified.

## AUTHOR CONTRIBUTIONS

**Mohammad M. Alnaeem**: Conceptualization; formal analysis; methodology; writing–original draft; writing–review and editing. **Ayman M. Hamdan‐Mansour**: Formal analysis; writing–original draft; writing–review and editing. **Abdulqadir J. Nashwan**: Writing–original draft; writing–review and editing. **Alaa Abuatallah**: Data curation; writing–original draft; writing–review and editing. **Mahmoud Al‐Hussami**: Data curation; writing–original draft; writing–review and editing.

## CONFLICTS OF INTEREST

The authors declare that they have no competing interests. Abdulqadir J. Nashwan is an Editorial Board member of Health Science Reports and coauthor of this article. He is excluded from editorial decision‐making related to the acceptance of this article for publication in the journal.

## ETHICS STATEMENT

The Institutional Review Board at Prince Hamza Hospital has approved this study. The current study followed the Declaration of Helsinki provisions, and all participants provided informed consent. The permission to use and translate copyrighted instruments has been received from the original author. No approval from hospitals was required according to the nature of the online study. The study was conducted following the EQUATOR research reporting checklist; (STROBE checklist) for cross‐sectional research. It was uploaded to the online system.

## TRANSPARENCY STATEMENT

The lead author Abdulqadir J. Nashwan affirms that this manuscript is an honest, accurate, and transparent account of the study being reported; that no important aspects of the study have been omitted; and that any discrepancies from the study as planned (and, if relevant, registered) have been explained.

## Data Availability

The authors confirm that the data supporting the findings of this study are available within the article.

## References

[1] Huang C , Wang Y , Li X , et al. Clinical features of patients infected with 2019 novel coronavirus in Wuhan, China. The Lancet,. 2020;395:497‐506. 10.1016/S0140-6736(20)30183-5 PMC7159299 31986264

[2] Kang L , Ma S , Chen M , et al. Impact on mental health and perceptions of psychological care among medical and nursing staff in Wuhan during the 2019 novel coronavirus disease outbreak: a cross‐sectional study. Brain Behav Immun. 2020;87:11‐17. 32240764 10.1016/j.bbi.2020.03.028 PMC7118532

[3] WHO. Coronavirus disease (COVID‐19) pandemic. 2020. Accessed September 13, 2022. https://www.who.int/emergencies/diseases/novel-coronavirus-2019

[4] Ashraf BN . Economic impact of government interventions during the COVID‐19 pandemic: international evidence from financial markets. J Behav Exp Finance. 2020;27:100371. 32835011 10.1016/j.jbef.2020.100371 PMC7323651

[5] Hoofman J , Secord E . The effect of COVID‐19 on education. Pediatr Clin North Am. 2021;68:1071‐1079. 34538299 10.1016/j.pcl.2021.05.009 PMC8445757

[6] Meyer BH , Prescott B , Sheng XS . The impact of the COVID‐19 pandemic on business expectations. Int J Forecast. 2022;38:529‐544. 35185229 10.1016/j.ijforecast.2021.02.009 PMC8846936

[7] Bauchner H , Fontanarosa PB , Livingston EH . Conserving supply of personal protective equipment—a call for ideas. JAMA. 2020;323:1911. 32196543 10.1001/jama.2020.4770

[8] Chen X , Zhang SX , Jahanshahi AA , et al. Belief in a COVID‐19 conspiracy theory as a predictor of mental health and well‐being of health care workers in Ecuador: cross‐sectional survey study. JMIR Public Health Surveill. 2020;6:e20737. 32658859 10.2196/20737 PMC7375774

[9] Firew T , Sano ED , Lee JW , et al. Protecting the front line: a cross‐sectional survey analysis of the occupational factors contributing to healthcare workers' infection and psychological distress during the COVID‐19 pandemic in the USA. BMJ Open. 2020;10:e042752. 10.1136/bmjopen-2020-042752 PMC7580061 33087382

[10] Shaukat N , Ali DM , Razzak J . Physical and mental health impacts of COVID‐19 on healthcare workers: a scoping review. Int J Emerg Med. 2020;13:40. 32689925 10.1186/s12245-020-00299-5 PMC7370263

[11] Zhang SX , Sun S . Developing and testing a measure of COVID‐19 organizational support of healthcare workers: results from Peru, Ecuador, and Bolivia. Psychiatry Res. 2020;291:113174. 32585436 10.1016/j.psychres.2020.113174 PMC7284268

[12] Barrett ES , Horton DB , Roy J , et al. Prevalence of SARS‐CoV‐2 infection in previously undiagnosed health care workers in New Jersey, at the onset of the U.S. COVID‐19 pandemic. BMC Infect Dis. 2020;20:20. 33198725 10.1186/s12879-020-05587-2 PMC7668027

[13] Hamdan‐Mansour A , Abdulla M , Khalifeh A , Hamaideh S , Alshibi A , Hamdan‐Mansour L . Determinants of healthcare workers' willingness to receive COVID‐19 vaccination. Malaysian J Med Health Sci. 2022;18:5‐13.

[14] Chemali Z , Ezzeddine FL , Gelaye B , et al. Burnout among healthcare providers in the complex environment of the middle east: a systematic review. BMC Public Health. 2019;19:1(19):1‐21. 31640650 10.1186/s12889-019-7713-1 PMC6805482

[15] Maslach C , Leiter MP . *The Truth About Burnout: How Organizations Cause Personal Stress and What to Do About It‏*. John Wiley & Sons; 2008.

[16] WHO *. Rational Use of Personal Protective Equipment for Coronavirus Disease (COVID‐19): Interim Guidance*; 2020.

[17] Dalky HF , Hamdan‐Mansour AM , Amarneh BH , et al. Social discrimination perception of health‐care workers and ordinary people toward individuals with COVID‐19. Soc Infl. 2020;15:65‐79‏.

[18] Nashwan AJ , Abujaber AA , Villar RC , Nazarene A , Al‐Jabry MM , Fradelos EC . Comparing the impact of COVID‐19 on nurses' turnover intentions before and during the pandemic in Qatar. J Pers Med. 2021;11:456. 34073655 10.3390/jpm11060456 PMC8225037

[19] Swamy L , Mohr D , Blok A , et al. Impact of workplace climate on burnout among critical care nurses in the veterans health administration. Am J Crit Care. 2020;29:380‐389. 32869073 10.4037/ajcc2020831

[20] Schwarzkopf D , Rüddel H , Thomas‐Rüddel DO , et al. Perceived nonbeneficial treatment of patients, burnout, and intention to leave the job among ICU nurses and junior and senior physicians. Crit Care Med. 2017;45:e265‐e273. 27776092 10.1097/CCM.0000000000002081

[21] Stone R , Wilhelm J , Bishop CE , Bryant NS , Hermer L , Squillace MR . Predictors of intent to leave the job among home health workers: analysis of The National Home Health Aide Survey. Gerontologist. 2017;57:890‐899. 27106825 10.1093/geront/gnw075

[22] Raso R , Fitzpatrick JJ , Masick K . Nurses' intent to leave their position and the profession during the COVID‐19 pandemic. J Nurs Adm. 2021;51:488‐494. 34519700 10.1097/NNA.0000000000001052

[23] Shiao JSC , Koh D , Lo LH , Lim MK , Guo YL . Factors predicting nurses' consideration of leaving their job during the SARS outbreak. Nurs Ethics. 2007;14:5‐17. 17334166 10.1177/0969733007071350

[24] Hamdan‐Mansour A , Alshibi A , Khalifa A , Hamdan‐Mansour L . Healthcare workers' knowledge and management skills of psychosocial and mental health needs and priorities of individuals with COVID‐19. Mental Health Soc Incl. 2020;24:135‐144.

[25] Al‐Hamdan Z , Manojlovich M , Tanima B , Alpha X . *Jordanian Nursing Work Environments, Intent to Stay, and Job Satisfaction*. Wiley Online Library‏. 10.1111/jnu.12265 27899008

[26] Newman M . Covid‐19: doctors' leaders warn that staff could quit and may die over lack of protective equipment‏. Br Med J. 2020;368:36. 10.1136/bmj.m1257 32217522

[27] Suleiman A , Bsisu I , Guzu H , et al. Preparedness of frontline doctors in Jordan Healthcare Facilities to COVID‐19 outbreak. Int J Environ Res Public Health. 2020;17(17):3181. 32370275 10.3390/ijerph17093181 PMC7246420

[28] Guo X , Wang J , Hu D , et al. Survey of COVID‐19 disease among orthopaedic surgeons in Wuhan, People's Republic of China. J Bone Joint Surg Am. 2020;102:847‐854. 32271208 10.2106/JBJS.20.00417 PMC7188039

[29] Varasteh S , Esmaeili M , Mazaheri M . Factors affecting Iranian nurses' intention to leave or stay in the profession during the COVID‐19 pandemic. Int Nurs Rev. 2022;69:139‐149. 34561862 10.1111/inr.12718 PMC8653279

[30] Hoseinabadi TS , Kakhki S , Teimori G , Nayyeri S . Burnout e seus fatores de influência entre enfermeiros de linha de frente e enfermeiros de outras enfermarias durante o surto de doença por coronavírus ‐COVID‐19‐ no irã. Invest Educ Enfer. 2020:38.

[31] Morgantini LA , Naha U , Wang H , et al. Factors contributing to healthcare professional burnout during the COVID‐19 pandemic: a rapid turnaround global survey. PLoS One. 2020;15(9):e0238217. 32881887 10.1371/journal.pone.0238217 PMC7470306

[32] Sung H , Ferlay J , Siegel RL , et al. Global cancer statistics 2020: GLOBOCAN estimates of incidence and mortality worldwide for 36 cancers in 185 countries. CA Cancer J Clin. 2021;71:209‐249. 33538338 10.3322/caac.21660

[33] Wan Z , Lian M , Ma H , Cai Z , Xianyu Y . Factors associated with burnout among Chinese nurses during COVID‐19 epidemic: a cross‐sectional study. BMC Nurs. 2022;21:51. 35227272 10.1186/s12912-022-00831-3 PMC8883459

[34] El‐Kurdy R , El‐Nemer A , Yousef A , Elsaidy W , Hamdan‐Mansour A . The moderation effect of affective commitment on the relationship between job stress and presenteeism among obstetric healthcare workers during COVID‐19 pandemic. Open Nurs J. 2022;16:16.

[35] Wu Y , Wang J , Luo C , et al. A comparison of burnout frequency among oncology physicians and nurses working on the frontline and usual wards during the COVID‐19 epidemic in Wuhan, China. J Pain Sympt Manage. 2020;60:e60‐e65. 10.1016/j.jpainsymman.2020.04.008 PMC7151285 32283221

[36] Chen HM , Liu CC , Yang SY , Wang YR , Hsieh PL . Factors related to care competence, workplace stress, and intention to stay among novice nurses during the coronavirus disease (COVID‐19) pandemic. Int J Environ Res Public Health. 2021;18:18. 10.3390/ijerph18042122 PMC7926418 33671613

[37] Yáñez JA , Jahanshahi AFSHAR , Alvarez‐Risco A , Li A J , Zhang SX . Anxiety, distress, and turnover intention of healthcare workers in Peru by their distance to the epicenter during the COVID‐19 crisis. Am J Trop Med Hyg. 2020;103:1614‐1620. 32815512 10.4269/ajtmh.20-0800 PMC7543861

[38] Rifin MATH , Danaee M . Association between burnout, job dissatisfaction and intention to leave among medical researchers in a research organisation in Malaysia during the COVID‐19 pandemic. Int J Environ Res Public Health. 2022;17:1729. 10.3390/ijerph191610017 PMC9407700 36011652

[39] Karimi L , Raei M , Parandeh A . Association between dimensions of professional burnout and turnover intention among nurses working in hospitals during coronavirus disease (COVID‐19) pandemic in Iran based on structural model. Front Public Health. 2022;10:860264. 35692308 10.3389/fpubh.2022.860264 PMC9174661

[40] Tabur A , Choudhury A , Emhan A , Mengenci C , Asan O . Clinicians' social support, job stress, and intent to leave healthcare during COVID‐19. Healthcare (Basel). 2022;10:10. 10.3390/healthcare10020229 PMC8872505 35206844

